# Depression trends in Europe: sociodemographic differences

**DOI:** 10.1093/eurpub/ckag168

**Published:** 2026-09-23

**Authors:** Johannes Beller, Julia Graßhoff, Stefanie Sperlich, Siegfried Geyer

**Affiliations:** Medical University Lausitz – Carl Thiem, Medical Sociology, Cottbus, Germany; Hannover Medical School, Medical Sociology, Hannover, Germany; Medical University Lausitz – Carl Thiem, Medical Sociology, Cottbus, Germany; Hannover Medical School, Medical Sociology, Hannover, Germany; Hannover Medical School, Medical Sociology, Hannover, Germany; Hannover Medical School, Medical Sociology, Hannover, Germany

## Abstract

Depression represents a critical concern for public mental health. This study examines time trends in the prevalence of elevated depressive symptoms throughout Europe and investigates socioeconomic differences. Data were derived from the European Social Survey waves of 2006, 2012, 2014, and 2023 (15 countries; *N* = 106 814). Prevalence of elevated depressive symptoms was assessed using the 8-item Center for Epidemiological Studies Depression Scale. Analyses were stratified by age, gender, education, income, occupation, limitation, and European region. Multilevel logistic regression models were used to analyze trends. Findings revealed substantial reductions over time in the prevalence of elevated depressive symptoms among older adults, smaller reductions in middle-aged individuals, and constant trends among the younger population. Considerable variations in depression trends were observed across most sociodemographic subgroups. Particularly beneficial trends were found in Eastern European middle-aged and older adults. Conversely, increasing trends among younger adults were mostly evidenced for Northern Europe, with similar patterns suggested for younger individuals with higher education and income. Additionally, those with limitations maintained an extremely high prevalence of elevated depressive symptoms over time, especially among younger individuals. Thus, while generally favorable trends were observed in older and middle-aged adults, increases in the prevalence of elevated depressive symptoms were found for parts of the younger population in Europe. Differential trends across sociodemographic groups were particularly present in terms of education, limitation, and geographic region. The findings highlight the necessity for targeted public health interventions addressing vulnerable groups. Further analyses are needed to identify intermediary determinants explaining these patterns.

## Introduction

Research consistently reveals substantial sociodemographic variations in depression prevalence, mirroring broader patterns of general morbidity [1–3]. Typically, individuals with lower income, lower education, and lower occupational status demonstrate a higher prevalence of depression compared to their more favored counterparts [1]. In addition to these socioeconomic status factors, differences in depression have been reported according to various other sociodemographic variables such as age, gender, urbanity, limitation and geographic region [4–7]. Understanding these disparities seems essential for developing effective prevention strategies and treatment approaches that address the needs of diverse populations. Therefore, sociodemographic differences need to be considered in studies on depression. At the population level, these patterns reflect how the social distribution of risk and protective conditions is structured across groups and contexts; as that distribution changes over time, the prevalence of depression in different population segments can change at different rates. Stratifying trends by age, gender, education, income, occupation, limitation, and region therefore allows us to assess whether population-level improvements are shared evenly or concentrated in particular segments.

Recently, researchers have focused on temporal patterns in depression prevalence, showing mixed findings regarding how depression is changing across different populations and regions. For example, Weinberger *et al.* [8] reported increasing depression trends among younger adults in the United States while noting more stable patterns among older adults. Similarly, Twenge *et al.* [9] documented substantial increases in depression symptoms among younger populations in the US. In a European context, Husky *et al.* [10] identified varying trends across occupational categories. Finally, Beller *et al.* [11] investigated changes in depressive symptoms in Europe and found a decrease in depressive symptoms. In the aforementioned study, the most pronounced improvements were observed among older adults, with moderate improvements in middle-aged adults, and minimal positive changes among younger adults. These varying results highlight the need for nuanced analyses of how depression trends might differ across sociodemographic groups.

Despite the growing body of research on mental health trends, significant gaps remain in our understanding of how depression patterns are evolving over time within and across sociodemographic groups in Europe [8, 11, 12]. This gap is particularly notable given the substantial social inequalities that have been observed in terms of depression [13]. Examining population trends, rather than prevalence at a single point in time, matters because the social conditions that shape mental health change across time and differ markedly between European regions; consequently, depression trends need not move in parallel across population groups, and inequalities between them may widen or narrow over time. Characterizing these differential population trends is a prerequisite for identifying potential vulnerable groups and for targeting prevention accordingly. Against this background, this study aims to examine time trends in the prevalence of elevated depressive symptoms across Europe, with specific emphasis on socioeconomic differences in these trends.

## Methods

### Sample

Data were drawn from the European Social Survey (ESS), a biennial cross-sectional survey providing comparative data on attitudes, beliefs, and behavior patterns across European populations [14]. Because our aim is to characterize population-based trends in the prevalence of elevated depressive symptoms rather than within-individual longitudinal trajectories, repeated cross-sectional surveys such as the ESS are well suited, as each wave draws a fresh, nationally representative sample and thereby captures changes in population composition over time without the potential selective attrition affecting panel studies. Data from all countries were used that participated across the four waves in which information on depressive symptoms was assessed (represented by ESS rounds 3, 6, 7, and 11, corresponding to years 2006, 2012, 2014, and 2023). The ESS uses population-based cross-sectional sampling of non-institutionalized participants aged 15 years and older, with interviews conducted mostly face-to-face in respondents’ residences. The final analytical sample included participants with complete responses from those 15 European countries that participated in all the previously mentioned ESS rounds (Belgium, Switzerland, Germany, Spain, Finland, France, United Kingdom, Hungary, Ireland, Netherlands, Norway, Poland, Portugal, Sweden, and Slovenia). Participants with missing values were deleted listwise (about 3.8% of the original sample, with missingness mostly resulting from the CES-D 8, 1.6% missing values, and occupation, 0.8% missing values). To assess whether this introduced bias, we regressed case exclusion on the key analytical variables. Exclusion was unrelated to education, but modestly more likely among non-working respondents and those reporting income difficulties (ORs ∼ 1.5–1.7). Given the small absolute share of excluded cases, these associations correspond to only minor absolute differences. Listwise deletion is therefore unlikely to introduce substantial bias, and more complex imputation procedures were not deemed necessary. Participants provided informed consent, and all procedures adhered to ethical standards in accordance with the 1964 Helsinki declaration and its later amendments. Since this study constitutes secondary analysis of anonymized data that had already been collected and is available for the scientific community, it is considered exempt from further ethical review according to guidelines governing research using existing datasets.

### Measures

Depression was measured using the 8-item version of the Center for Epidemiologic Studies Depression Scale (CES-D 8) [15]. First, a total depression score was calculated as the sum of all responses (range = 0–24), with higher scores indicating more severe depressive symptoms. Finally, in accordance with the literature the prevalence of elevated depressive symptoms was operationalized as a dichotomous score using a cut-off of equal to or greater than 9 [16]. Previous studies have confirmed the psychometric validity of the CES-D 8 [17, 18].

Sociodemographic variables included age, which was also categorized into three groups for stratification: younger (≤39 years), middle-aged (40–65 years), and older (66+ years). Gender was coded as male or female. Occupational status was classified into the five categories “high-skilled white-collar,” “low-skilled white-collar,” “high-skilled blue-collar,” “low-skilled blue-collar,” and “non-working” based on the self-reported occupational status in accordance with the International Standard Classification of Occupations (White-Collar High-Skilled: ISCO 1, 2, 3; White-Collar Low-Skilled: ISCO 4, 5; Blue-Collar High-Skilled: ISCO 6, 7; Blue-Collar Low-Skilled: ISCO 8, 9; and Non-Working). Educational attainment was classified according to the International Standard Classification of Education (ISCED) and categorized as low (ISCED I, II corresponding to primary or lower secondary education), intermediate (ISCED III, IV corresponding to higher secondary education), or high (ISCED V and VI corresponding to tertiary education). Income was measured as the respondent’s assessment about the household’s current income with responses categorized as “comfortable” (living comfortably on present income), “coping” (coping on present income), and “difficult” (finding it difficult or very difficult on present income). Limitation was measured by responses to whether participants reported to be hampered in their daily activities by a health condition, with responses dichotomized as limited (in case participants responded with “yes, a lot” or “yes, to some extent”) or not limited. Urbanity was classified as participants living in urban areas (including big cities, suburbs or outskirts of big cities, towns, or small cities) or rural areas (country villages, farms, or homes in countrysides). European regions were categorized in accordance with the United Nations geo-scheme as North (Ireland, Finland, Norway, Sweden, United Kingdom), West (Belgium, France, Germany, Netherlands, Switzerland), East (Hungary, Poland), and South (Portugal, Slovenia, Spain).

### Data analysis

First, descriptive statistics of all variables are reported based on the unweighted data to describe the analytical sample across time periods. Then, to determine population trends, weighted multilevel logistic regression analyses are conducted predicting depression using age (in years), gender (0 = male; 1 = female) and time period (scaled fractionally as a metric variable between 0 and 1 with 2006 = 0, 2012 = 0.35, 2014 = 0.47, and 2023 = 1, such that the effect of time period can be interpreted as the average change between 2006 and 2023). In the overall association model, time period was additionally entered as a categorical predictor (with 2006 as the reference) to permit a test of the linearity assumption; all trend and subgroup models used the fractionally scaled metric coding described above. We estimated multilevel logistic regression models in which individual respondents (level 1) were nested within countries (level 2), specifying random intercepts for countries. Random slopes for time period were not included, because the small number of level-2 units (15 countries) does not permit their stable estimation. In the overall model, the country-level intercept variance was 0.055 (SD = 0.235), corresponding to an intraclass correlation of 0.016. Thus, only about 1.6% of the total variation in depressive symptoms was located at the country level, with the remainder attributable to individual-level differences. Model fit indices for the overall model were AIC = 84 284, BIC = 84 485, and log-likelihood = −42 121. All regression analyses, including the subgroup analyses, were weighted and controlled for age and gender.

Trends were also analyzed in stratified samples according to sociodemographic covariates. To formally test whether trends differed significantly between sociodemographic groups, additional multilevel logistic regression analyses were conducted including interaction terms between time period and the respective sociodemographic variable. In addition to the widely used odds ratios, predictive probabilities are also calculated based on multilevel logistic regression analyses, directly comparing the predicted prevalence of depression in 2006 with 2023.

## Results

As seen in Table 1, the overall prevalence of depression across all time periods was 19.23%, declining from 21.47% in 2006 to 17.73% in 2023. Over the same period, the sample grew older (mean age: 47.85 to 51.20 years; older adults 66+: 19.84% to 26.67%), while socioeconomic conditions improved, with higher education rising from 25.17% to 30.23% and comfortable income from 33.71% to 42.35%.

**Table 1:** Sociodemographic characteristics.^a^

	Stratified by time period				
	Overall	2006	2012	2014	2023
*N*	106 814	27 681	28 764	26 770	23 599
Depression (%)	19.23	21.47	19.85	17.57	17.73
Age [mean (SD)]	49.30 (18.69)	47.85 (18.47)	48.85 (18.59)	49.61 (18.67)	51.20 (18.89)
Age-group (%)					
≤39	33.45	36.22	34.06	32.79	30.19
40–65	44.05	43.94	44.62	44.36	43.15
66+	22.50	19.84	21.32	22.85	26.67
Female (%)	52.78	53.75	52.95	52.16	52.13
Education (%)					
High	24.29	25.17	20.21	22.54	30.23
Intermediate	44.57	38.91	46.32	46.75	46.62
Low	31.13	35.92	33.47	30.71	23.15
Income (%)					
Comfortable	35.25	33.71	30.69	35.49	42.35
Coping	45.36	47.17	45.49	45.35	43.10
Difficult	19.39	19.13	23.82	19.15	14.56
Occupation (%)					
WC-HS	25.65	24.02	23.66	25.33	30.35
WC-LS	12.82	13.71	12.71	12.46	12.31
BC-HS	6.74	7.72	6.29	6.75	6.15
BC-LS	7.39	8.12	7.43	6.85	7.08
Non-working	47.40	46.43	49.91	48.61	44.11
Health limitation (%)	26.25	25.36	25.57	26.72	27.59
Rural (%)	39.47	39.79	37.80	38.75	41.96
Region (%)					
North	34.06	33.55	35.29	35.66	31.33
West	35.97	36.47	34.31	37.05	36.20
East	12.24	10.87	12.69	11.56	14.08
South	17.73	19.10	17.71	15.74	18.39

a Unweighted estimates.

Abbreviations: BC-HS = blue-collar high skilled; BC-LS = blue-collar low skilled; N = sample size; SD = standard deviation; WC-HS = white-collar high skilled; WC-LS = white-collar low skilled.

As seen in Table 2, the multilevel logistic regression analysis revealed several significant sociodemographic differences in depression. The three most strongly associated factors were financial difficulty (OR = 3.91, 95% CI [3.72; 4.12]), limitations (OR = 3.44, 95% CI [3.32; 3.57]), and being female (OR = 1.52, 95% CI [1.47; 1.57]; all *P* < .001). Because time period was entered as a categorical predictor (Ref. 2006), Table 2 also serves as a test of the linearity assumption: all later survey years showed significantly lower odds of depression than 2006, but the decline was non-linear, reaching a low in 2014 (OR = 0.77, 95% CI [0.74; 0.81]) before partially rebounding in 2023 (OR = 0.88, 95% CI [0.84; 0.92]). Eastern and Southern European regions showed significantly higher odds of depression than Northern Europe (OR = 1.89 and OR = 1.68, respectively); low educational attainment and non-working status were also associated with increased odds, albeit with smaller effect sizes.

**Table 2:** Sociodemographic and socioeconomic associations with elevated depressive symptoms across Europe (2006–2023): odds ratios from multilevel logistic regression.

	OR	95% CI	*P*
Time period			
2006 (Ref.)	–	–	–
2012	0.84	[0.8; 0.88]	<.001
2014	0.77	[0.74; 0.81]	<.001
2023	0.88	[0.84; 0.92]	<.001
Age-group			
≤39 (Ref.)	–	–	–
40–65	1.03	[0.99; 1.07]	.174
66+	0.96	[0.92; 1.02]	.165
Gender (female)	1.52	[1.47; 1.57]	<.001
Occupation			
WC-HS (Ref.)	–	–	–
WC-LS	1.11	[1.04; 1.18]	.003
BC-HS	1.04	[0.95; 1.13]	.388
BC-LS	1.27	[1.17; 1.37]	<.001
Non-working	1.37	[1.29; 1.45]	<.001
Education			
High (Ref.)	–	–	–
Intermediate	1.12	[1.07; 1.18]	<.001
Low	1.39	[1.31; 1.47]	<.001
Income			
Comfortable (Ref.)	–	–	–
Coping	1.61	[1.54; 1.69]	<.001
Difficult	3.91	[3.72; 4.12]	<.001
Health limitation (yes)	3.44	[3.32; 3.57]	<.001
Urbanity (rural)	0.83	[0.80; 0.86]	<.001
Region			
North (Ref.)	–	–	–
West	1.19	[0.89; 1.6]	.242
East	1.89	[1.28; 2.79]	.001
South	1.68	[1.19; 2.35]	.003

Abbreviations: BC-HS = blue-collar high skilled; BC-LS = blue-collar low skilled; WC-HS = white-collar high skilled; WC-LS = white-collar low skilled.

### Time trends

**Figure 1:**
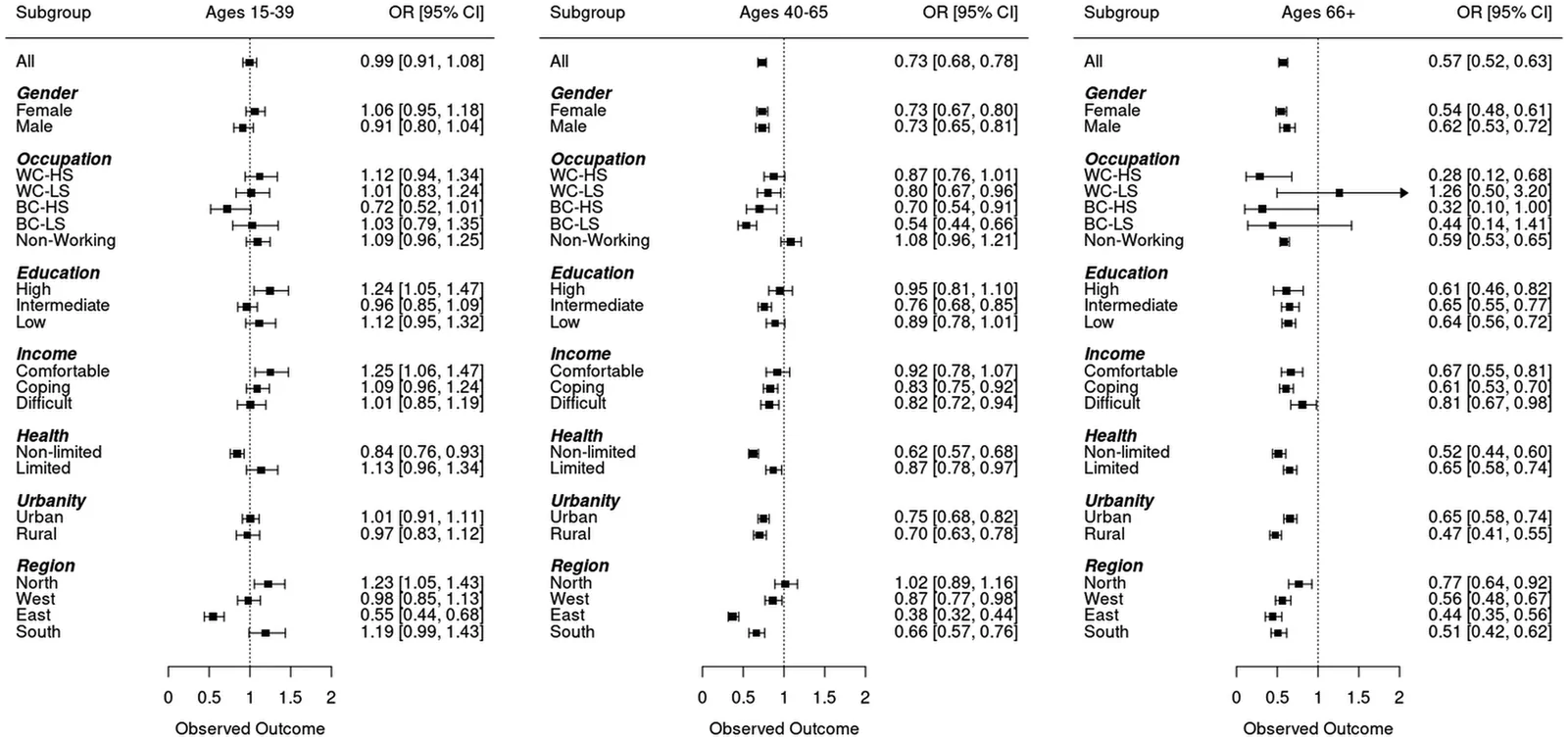
Trends in elevated depressive symptoms (2006–2023) by sociodemographic subgroup: odds ratios for time period from age- and gender-adjusted multilevel logistic regression. Notes. Depicted are the odds ratios for time period predicting depression controlling for age and gender via multilevel logistic regression analyses using weights. For each subgroup a separate multilevel logistic regression analysis was conducted. Abbreviations: BC-HS, blue-collar high skilled; BC-LS, blue-collar low skilled; WC-HS, white-collar high skilled; WC-LS, white-collar low skilled.

**Figure 2:**
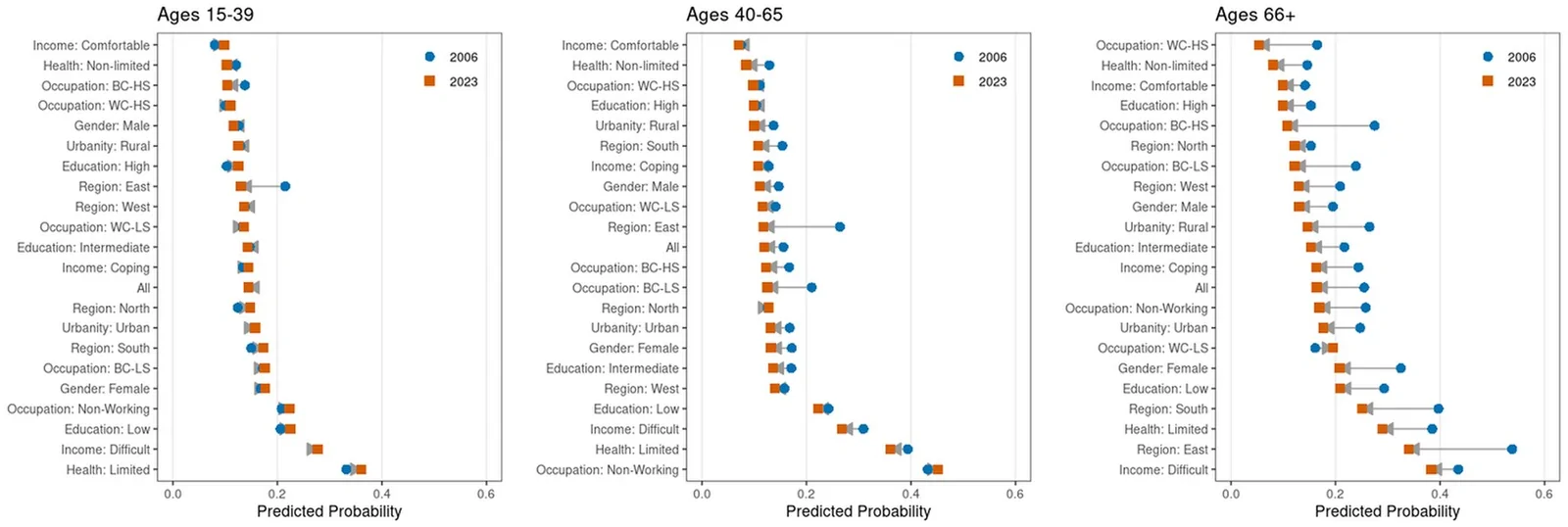
Predicted prevalence of elevated depressive symptoms in 2006 versus 2023 by sociodemographic subgroup, from age- and gender-adjusted multilevel logistic regression. Notes. Depicted are model-based predicted probabilities from the fitted multilevel logistic regression models, obtained by setting the time variable to the first (2006) versus the last (2023) survey round and generating population-average predictions on the response scale, with random effects omitted. For each subgroup a separate weighted multilevel logistic regression analysis was conducted. Predictions were calculated at fixed reference values for the adjustment variables, using the mean age of the respective age groups and a balanced reference coding for gender; they therefore represent predictions at specified covariate values rather than average marginal effects. Confidence intervals are not shown in Fig. 2 because including them for all subgroup estimates would have substantially reduced readability and made the figure difficult to interpret visually. To ensure transparency about uncertainty, the corresponding regression results report confidence intervals (Fig. 1). Abbreviations: BC-HS, blue-collar high skilled; BC-LS, blue-collar low skilled; WC-HS, white-collar high skilled; WC-LS, white-collar low skilled.

As depicted in Figs 1 and 2, multilevel logistic regression and predicted probability analyses were conducted to study depression trends in the three age groups and across socioeconomic subgroups.

For younger adults (ages 15–39), the overall odds ratio for time period was 0.99 [0.91, 1.08], indicating essentially no change over time. However, trends differed across sociodemographic factors, with significant interactions between time period and limitation (*P* = .002) and region (*P* < .001); the interaction with education did nearly reach significance (*P* = .088); however, given the large number of subgroup tests, it is interpreted only tentatively. Depression odds increased significantly over time among those with high education (OR = 1.24 [1.05, 1.47]), in Northern Europe (OR = 1.23 [1.05, 1.43]), and among those with comfortable income (OR = 1.25 [1.06, 1.47]), whereas they decreased in Eastern Europe (OR = 0.55 [0.44, 0.68]) and among those without limitations (OR = 0.84 [0.76, 0.93]). Accordingly, the overall predicted probability of depression remained essentially unchanged at 14.5% between 2006 and 2023, but increased among those with high education (10.3% to 12.5%) and in Northern Europe (12.4% to 14.8%), while decreasing substantially in Eastern Europe (21.5% to 13.0%). Notably, young adults with health limitations maintained the highest predicted probability of depression throughout, rising from 33.2% to 36.0%. Other groups with particularly high probabilities in 2023 included those with income difficulties (27.7%), low education (22.5%), and non-working individuals (22.3%).

For middle-aged adults (ages 40–65), the overall odds ratio for time period was 0.73 [0.68, 0.78], indicating a significant decrease in depression prevalence between 2006 and 2023. Significant interactions emerged between time period and occupation, limitation, region (all *P* < .001), and education (*P* = .029). Decreases over time were particularly strong among workers in blue-collar low skilled occupations (OR = 0.54 [0.44, 0.66]), in Eastern Europe (OR = 0.38 [0.32, 0.44]), and among those without limitations (OR = 0.62 [0.57, 0.68]). Accordingly, the overall predicted probability of depression decreased from 15.6% to 11.9%, with the most substantial improvements in Eastern Europe (26.4% to 11.8%) and among blue-collar workers with low skills (21.0% to 12.5%). Despite these positive trends, non-working individuals (45.2%), those with limitations (36.1%), and those with income difficulties (26.8%) maintained high depression probabilities in 2023.

For older adults (ages 66+), the overall odds ratio for time period was 0.57 [0.52, 0.63], indicating a substantial and significant decrease in depression prevalence. Significant interactions emerged between time period and income (*P* = .032), occupation (*P* = .030), urbanity (*P* = .002), limitation (*P* = .018), and region (*P* = .003). Decreases over time were particularly strong among older workers in white-collar high skilled occupations (OR = 0.28 [0.12, 0.68]), rural residents (OR = 0.47 [0.41, 0.55]), Eastern Europeans (OR = 0.44 [0.35, 0.56]), and individuals without limitations. Accordingly, the overall predicted probability of depression decreased from 25.5% to 16.4%, with the most substantial improvements among white-collar high skilled (16.5% to 5.3%) and blue-collar high skilled occupations (27.5% to 10.7%), rural residents (26.5% to 14.6%), and those without limitations (14.6% to 8.1%). Despite these positive trends, those with income difficulties maintained a high depression probability in 2023 (38.3%, down from 43.5%).

## Discussion

We found substantial depression trends over time that differ strongly by sociodemographic characteristics: our analyses revealed a consistent pattern of improvement among older adults (66+) with a substantial decrease in the odds of elevated depressive symptoms, and a moderate improvement among middle-aged adults (40–65). In contrast, younger adults (15–39) showed stable trends on average. However, these age-specific patterns were further moderated by socioeconomic factors, with particularly striking improvements among all Eastern Europeans and those without limitations. On the other hand, increasing depression trends emerged for younger Northern Europeans, younger Europeans with a comfortable income, and younger Europeans with a higher educational attainment. Furthermore, across all age groups, those with limitations maintained among the highest depression prevalence of all subgroups. Thus, depression trends are not uniform but highly depend on the specific socioeconomic subgroup considered.

Our results both align with and extend beyond previous research on depression trends across different population subgroups. Our findings align with Weinberger *et al.*and Twenge *et al.* regarding some concerning increases in depression among younger adults, while also supporting Beller *et al.* who found improvements in depressive symptoms among older adults in Europe [8, 9, 11, 12]. However, our study extends this knowledge by showing that sociodemographic subgroups experience dramatically different depression trends. For example, the substantial improvements we observed in Eastern Europe compared to other regions suggest that regional developmental patterns may be an important determinant for mental health trends [7]. The data furthermore reveal that trends in depression among younger adults depend on the socioeconomic status, with e.g. those with a higher educational attainment possibly emerging as a more vulnerable subgroup over time rather than a protective one, although the underlying education × time interaction did not reach statistical significance and this pattern should therefore be regarded as tentative [19]. This reduces traditional socioeconomic gradients in mental health and challenges conventional wisdom about education’s global protective effects for mental health [20]. Finally, limitation prevalence has been reported to increase strongly among younger cohorts, and it has been speculated in the literature that this increase would be accompanied by a reduced psychosocial burden [21]; however, the current results suggest that the contrary might be true: the depressive symptom burden of those with limitations seems to have remained the same or even increased over time in the younger population in contrast to those without limitations. In summary our research provides a more nuanced depiction of temporal mental health patterns than previously documented by identifying dramatically different depression trends across sociodemographic subgroups.

### Implications

From a public health perspective, our findings present both encouraging and concerning trends. The substantial reductions in depression among older and middle-aged adults represent a significant public health achievement, potentially reflecting successful social policies, improved healthcare access, and economic development across much of Europe [3, 22]. However, the increasing depression prevalence among many younger individuals, especially those with higher socioeconomic status, signals an emerging public health crisis requiring public health attention.

This dual pattern of improvement in older adults and stagnation/deterioration in younger cohorts also has important theoretical implications related to theories on morbidity development, like the compression of morbidity hypothesis [23, 24]. While older adults appear to be experiencing a compression of “psychological morbidity”—aligning with Fries’ hypothesis that illness is being compressed into a shorter period at the end of life—younger adults may be facing an expansion of morbidity, with health problems emerging earlier in life and potentially persisting longer [25, 26]. This pattern aligns with previous research documenting worsening functional and physical health indicators among younger generations [12, 27, 28]. Thus, taken together, the results of the current study and the literature suggests a “double development of morbidity,” with compressing and expanding morbidity occurring at the same time but in different age-cohorts. Further studies are needed integrating insights from social determinants research with life-course epidemiology to develop age-appropriate and contextually sensitive explanations for the observed trends [29, 30].

### Limitations and future research directions

Several limitations warrant consideration when interpreting the findings. First, the study relies on self-reported depression symptoms using the CES-D scale, that, although standardized and validated, may not perfectly align with clinical diagnoses. However, this approach offers the advantage of also capturing trends in the at-risk population. Second, while the ESS provides representative coverage, response rates have declined over time in some countries, potentially introducing health selection bias [31, 32]. Treating time period categorically further showed that the overall decline in depressive symptoms was not strictly linear, with a partial rebound between 2014 and 2023. The survey waves are unequally spaced, and the interval between 2014 and 2023 spans the onset of the COVID-19 pandemic. In the absence of a measurement around 2019, the observed changes cannot be unambiguously attributed to a long-term secular development as opposed to a more acute, pandemic-related effect, and the precise timing of these changes within this interval cannot be established. Nevertheless, this dataset’s consistent methodology and multi-wave structure represent a considerable strength for examining time trends. Third, while multiple sociodemographic factors were considered, unmeasured intermediary variables such as sedentary lifestyle, sleep patterns and pandemic-related stressors could provide additional explanatory power for the observed trends, particularly among younger adults [33]. Fourth, because the analyses are based on repeated cross-sectional samples, individuals observed within a given age group at the start of the study period belong to different birth cohorts than those observed in the same age group at its end. The observed trends therefore reflect a combination of period, cohort, and compositional effects that cannot be fully disentangled with these data; while a full age-period-cohort analysis was beyond the scope of the present study, this ambiguity should be borne in mind when interpreting the age-specific trends, particularly among younger adults. A final limitation concerns the possibility of selective non-response and selective item missingness. Individuals with more severe depressive symptoms, as well as vulnerable subgroups such as non-working respondents and those reporting income difficulties, were somewhat more likely to be excluded. Because these are higher-risk groups, the prevalence of depression and the extent of social inequalities are, if anything, underestimated; any resulting bias in the subgroup analyses is therefore modest and conservative in direction. Despite these limitations, we are able to document important sociodemographic variations in depression trends across Europe, providing a comprehensive analysis that integrates age, socioeconomic characteristics, and regional context in understanding mental health trends. Future research should build upon our documented sociodemographic variations by exploring the underlying mechanisms driving these divergent trends, particularly focusing on the deteriorating mental health among younger Europeans.

## Data Availability

The data underlying this article are available from the ESS Data Portal, at https://ess.sikt.no/en/. Key points Prevalence of elevated depressive symptoms decreased significantly in Europe between 2006 and 2023, particularly among older adults and middle-aged adults, while stagnating among younger adults Substantial sociodemographic differences in depression trends were observed, with Eastern Europeans experiencing especially pronounced reductions and some younger adults with a higher socioeconomic status experiencing increases in the prevalence of elevated depressive symptoms Individuals with health limitations maintained high prevalence of elevated depressive symptoms throughout the study period, highlighting persistent inequalities despite overall population improvements Public health attention should focus on emerging vulnerable groups, particularly younger adults with higher socioeconomic status, while maintaining successful approaches that have benefited older populations and Eastern Europeans
